# Polysaccharides from *Pleurotus eryngii*: sequential extraction and structural characterization

**DOI:** 10.1016/j.fochx.2026.103543

**Published:** 2026-01-20

**Authors:** Ziyan Sun, Zhicong Song, Andong Zhou, Yongjian Lin, Jiahui Yao, Kevin H. Mayo, Yifa Zhou, Lin Sun

**Affiliations:** a Engineering Research Center of Glycoconjugates of Ministry of Education, Jilin Provincial Key Laboratory of Chemistry and Biology of Changbai Mountain Natural Drugs, School of Life Sciences, Northeast Normal University, Changchun 130024, China; b Department of Biochemistry, Molecular Biology & Biophysics, University of Minnesota, 6-155 Jackson Hall, Minneapolis, MN 55455, USA

**Keywords:** *Pleurotus eryngii*, Polysaccharide, Sequential extraction, Glucan, Galactan, Mannan

## Abstract

Polysaccharides, a primary component from *Pleurotus eryngii* (*P. eryngii*), have diverse biological activities. To systematically investigate structural features of these polysaccharides, we have developed a sequential extraction strategy with gradient-enhanced solvent-oriented purification that includes cold and hot water, enzymes assisted, dilute and concentrated alkali. Five polysaccharides CWP (0.19%), HWP (0.1%), EAP (0.14%), DAP (0.65%), CAP (0.34%) were obtained. The sequential extraction strategy developed here is practical, significantly improving the total extraction yield compared to single methods. After purification, we obtained eight homogeneous polysaccharides. Using several characterization techniques, we discovered that *P. eryngii* polysaccharides contained five structural types, including β-1,3-D-glucan, β-1,6-D-glucan, α-1,6-D-galactan, α-1,3-D-mannan, and a novel polysaccharide α-1,2-D-mannan, with ratios of 20:7:4.3:1.6:1. This study provides valuable insights into the structural diversity and relative abundance of polysaccharides in *P. eryngii*. Our findings provide a foundation for further investigation into their structure-activity relationships and potential applications.

## Highlights

• A sequential extraction method was developed for efficient extraction *Pleurotus eryngii* polysaccharides.
• *Pleurotus eryngii* polysaccharides contained β-1,3/1,6-D-glucan, α-1,6-D-galactan and α-1,3/1,2-D-mannan.
• The mass ratios of five types of polysaccharides in *Pleurotus eryngii* was 20:7:4.3:1.6:1.
• α-1,2-D-mannan was first identified in *Pleurotus eryngii*.

## 1 Introduction

Polysaccharides derived from edible mushrooms are widely available and exhibit a variety of biological activities (Helena et al., 2024), making them promising resources for food and pharmaceutical development. These polysaccharides are currently the focus in research and development of health foods and drugs. Edible mushroom polysaccharides have diverse structures and can be classified into three main types based on their monosaccharide composition: glucans (Yang et al., 2024), galactans (Zavadinack et al., 2021), and mannans (Anna et al., 2016). This structural diversity, including molecular weight, monosaccharide composition, types of glycosidic linkages, and arrangement of side chains, endows them with multiple biological activities, such as maintaining intestinal health (Li et al., 2024), anti-cancer effects (Lara et al., 2025), antioxidant properties (Wang et al., 2015), neuroprotection (Zhou et al., 2023), and the ability to lower blood glucose and lipid levels.

*P. eryngii* has a distinctive almond-like aroma and abalone-like texture. It possesses high nutritional value, being rich in proteins and vitamins. As a primary active component, its polysaccharides have complex and diverse structures, including various types, such as glucans (Wang et al., 2022) and galactans (Abreu et al., 2021). *P. eryngii* polysaccharides are reported to display diverse biological activities, including immunomodulatory effects, antitumor activity, antioxidant properties, hypoglycemic effects, and hypolipidemic properties. Currently, various extraction techniques have been employed to obtain polysaccharides from *P. eryngii*, including hot water extraction (Ma et al., 2014), alkali extraction (Ren et al., 2017), as well as enzyme-assisted extraction and microwave-assisted extractions (Leong et al., 2021). Generally, a single extraction approach cannot sufficiently extract all types of polysaccharides from mushrooms. Furthermore, some extractions result in a mixture of polysaccharides with different structural types, making it difficult for their separation and purification, which hinders structural analyses. Studies have shown that β-1,3-D-glucan from *P. eryngii* is characterized by a main chain composed of β-1,3-linked Glc*p* residues, with t-β-D-Glc*p* side chains attached at the O-6 position of glucose units (Tao et al., 2025). β-1,6-D-glucan features a backbone of β-1,6-linked Glc*p* residues, bearing t-β-D-Glc*p* side chains at the O-3 position (Lim et al., 2008). Galactans on the other hand, primarily possess a main chain of α-1,6-linked D-Gal*p* residues, with side chains of t-β-D-Man*p* attached at the C-2 position (Yang et al., 2013). Currently, there are few reports on mannans from *P. eryngii*, making its structural characterization unclear.

Despite the significance of biological activities of polysaccharides from *P. eryngii*, current extraction approaches are insufficient to structurally investigate these polysaccharides. The specific structural types and their relative contents have not been fully elucidated, which hinders in-depth investigations into their structure-activity relationships and limits the application of these polysaccharides. This study was aimed to develop a sequential extraction method to efficiently extract and purify different types of polysaccharides from *P. eryngii*. Our comprehensive approach utilizes multiple techniques for the separation and purification of these polysaccharides to obtain homogeneous fractions that can be systematically characterized in terms of their structural features. These findings provide new directions and strategies for the extraction of polysaccharides from *P. eryngii* and demonstrate their structural diversity. Our results have established a foundation for further investigations into their structure-activity relationships.

## 2 Materials and methods

### 2.1 Materials

Fresh fruiting bodies of *P. eryngii* were purchased from the local market and identified by rDNA-ITS sequencing analysis. Standards, including mannose (Man), glucose (Glc), galactose (Gal), rhamnose (Rha), fucose (Fuc), xylose (Xyl), arabinose (Ara), galacturonic acid (GalA), glucuronic acid (GlcA), were obtained from Sigma (St. Louis, MO, USA). Methylated-galactose (Me-Gal) was prepared in our lab. All other chemicals and reagents were of analytical grade and made in China.

### 2.2 Extraction of polysaccharides from *P. eryngii*

Cold-water extraction: 10 kg of fresh *P. eryngii* fruiting bodies were mixed with 40 L of deionized water (dH_2_O, 1:4 *w*/*v*), soaked at room temperature for 24 h, and filtered through cheesecloth. The filtrate volume was vacuum reduced to 3 L at 60 °C and centrifuged (4500 rpm, 10 min). Anhydrous ethanol (60% *v*/v) was added to the supernatant for precipitation. After further centrifugation (4500 rpm, 15 min), the precipitate was collected and redissolved in 1 L dH_2_O, dialyzed (3500 Da, 24 h), and vacuum freeze-dried to obtain the cold water extracted polysaccharide (CWP).

Hot-water extraction: The residue from the cold-water extraction was mixed with distilled water at a 1:4 (*w*/*v*) solid-to-liquid ratio (total 40 L), extracted at 100 °C for 3 h, and filtered to collect the extract. The same extraction process was repeated again, and extracts were combined, concentrated to 3 L, and centrifuged (4500 rpm, 10 min). Anhydrous ethanol was added to the supernatant to a final concentration of 60% (*v*/v) for polysaccharide precipitation at room temperature. After centrifugation (4500 rpm, 15 min), the precipitate was redissolved in 1 L distilled water, dialyzed with a 3500 Da membrane for 24 h, and vacuum freeze-dried to yield the hot water extracted polysaccharide (HWP).

Enzyme-assisted extraction: The residue from hot-water extraction was mixed with distilled water at a 1:4 (*w*/*v*) solid-to-liquid ratio (total 40 L). Cellulase, α-amylase, and glucosidase were added at 1% of the mass, followed by extraction at 50 °C for 3 h. The mixture was then filtered through gauze. The extract was inactivated at 100 °C for 10 min, vacuum-concentrated to 3 L, and centrifuged (4500 rpm, 15 min). The supernatant was dialyzed with a 3500 Da membrane under running water for 24 h, and vacuum freeze-drying was performed to yield the enzyme-assisted extracted polysaccharide (EAP).

Dilute-alkali extraction: The residue from enzyme-assisted extraction was mixed with 18 L of 0.5 M NaOH solution at a 1:1.8 (*w*/*v*) solid-to-liquid ratio, extracted at 80 °C for 3 h, and filtered. The same procedure was repeated. Extracts were combined and neutralized at pH 7 with concentrated hydrochloric acid. The mixture was vacuum-concentrated to 3 L, centrifuged (4500 rpm, 10 min), and the supernatant was dialyzed with a 3500 Da membrane for 48 h (continuous dialysis). Finally, vacuum freeze-drying was conducted to yield the dilute-alkali extracted polysaccharide (DAP).

Concentrated-alkali extraction: The residue from dilute-alkali extraction was mixed with 10 L of 1.25 M NaOH solution at a 1:1 (*w*/*v*) solid-to-liquid ratio, soaked at room temperature for 3 h, and filtered to collect the extract. This procedure was repeated. Extracts were combined and neutralized at pH 7 with concentrated hydrochloric acid. The mixture was vacuum-concentrated to 2 L, centrifuged (4500 rpm, 10 min), and the supernatant was dialyzed with a 3500 Da membrane for 48 h (continuous dialysis). Vacuum freeze-drying was performed to yield the concentrated-alkali extracted polysaccharide (CAP).

### 2.3 Separation and purification of polysaccharides

500 mg of CWP was dissolved in 10 mL of 0.15 M NaCl, vortexed, and centrifuged at 12,000 rpm for 5 min. The supernatant was collected and loaded onto a pre-equilibrated Bestarose CL-6B column (2.6 × 100 cm, Bestchrom, China), eluted with 0.15 M NaCl solution at a flow rate of 0.4 mL/min, and fractions were collected every 20 min (one tube per fraction). Sugar content in each eluate tube was determined using the phenol‑sulfuric acid method. Elution peaks were labeled accordingly, and eluates of the same fraction were combined. The combined fractions were dialyzed and freeze-dried to yield two homogeneous fractions CWP-a and CWP-b. 

2 g of HWP was dissolved in 200 mL deionized water (dH_2_O). Absolute ethanol was slowly added to the solution to a final concentration of 40% (*v*/v), followed by standing overnight at 4 °C and centrifugation (12,000 rpm, 15 min). The supernatant and precipitate were collected separately, with ethanol removed through freeze-drying. One gram of the resulting precipitate was dissolved in 100 mL dH_2_O. Absolute ethanol was again added to 30% (v/v) final concentration. The mixture stood at 4 °C overnight, centrifuged (12,000 rpm, 15 min), and the precipitate was collected, with ethanol removed by freeze-drying to yield fraction HWP-a. Three hundred milligrams of the 40% ethanol treatment supernatant was dissolved in 15 mL dH_2_O, vortexed, centrifuged (12,000 rpm, 15 min), and the supernatant was loaded onto a pre-equilibrated 50 mL DEAE-Bestarose Fast Flow ion exchange column. The column was first eluted with 200 mL dH_2_O and then with 0.5 M NaCl at 0.5 mL/min. The NaCl eluted fractions were dialyzed and freeze-dried to yield HWP-b.

500 mg of EAP was dissolved in 10 mL of 0.15 M NaCl solution and centrifuged at 12,000 rpm for 5 min. The supernatant was collected and loaded onto a pre-equilibrated Chromedex 75 gel column (2.6 × 100 cm, Bestchrom, China), eluted with 0.15 M NaCl at a flow rate of 0.4 mL/min, with fractions collected every 20 min. Sugar content in each tube was determined using the phenol‑sulfuric acid method, yielding the purified fraction EAP-a.

60 g of DAP was dissolved in 3 L deionized water (dH_2_O), filtered through 800-mesh gauze, and separated using a 0.2 μm ceramic membrane ultrafiltration system at a constant 0.2 MPa. The retentate was concentrated, and absolute ethanol was added to 40% (*v*/v) for precipitation at room temperature. After centrifugation (4500 rpm, 20 min), the precipitate was collected and freeze-dried to yield the fraction DAP-a. Three hundred milligrams of the permeate was dissolved in 15 mL dH_2_O, centrifuged at 12,000 rpm for 15 min, and the supernatant was loaded onto a pre-equilibrated 50 mL DEAE-Bestarose Fast Flow column (2.6 × 10 cm, Bestchrom, China). The column was first eluted with 200 mL dH_2_O, followed by elution with 0.5 M NaCl at 0.5 mL/min. The NaCl eluted fractions were dialyzed and freeze-dried to yield fraction DAP-b.

1 g of CAP was dissolved in 100 mL deionized water (dH_2_O) until fully dissolved. Absolute ethanol was added to a final concentration of 40% (*v*/v), and the mixture was stored at 4 °C for polysaccharide precipitation. After centrifugation at 12,000 rpm for 15 min, the precipitate was collected and freeze-dried to yield purified fraction CAP-a.

### 2.4 Total carbohydrate, uronic acid, protein and ash content determination

The sugar content of each polysaccharide fraction was determined by using the phenol‑sulfuric acid method, using a mixture of the primary monosaccharides in each polysaccharide fraction as the reference standard (Dou, Hu, & Kang, 2025). The uronic acid content was determined using the *m*-hydroxydiphenyl method with GlcA as the reference standard (Blumenkrantz & Asboe-Hansen, 1973). The protein content was determined using the Coomassie Brilliant Blue method with bovine serum albumin (BSA) as the standard (Sedmak & Grossberg, 1977). The ash content was measured by heating the polysaccharide in a muffle furnace at 550 °C for 5 h until the sample reached a constant weight (Sang et al., 2010).

### 2.5 Molecular weight determination

Molecular weight determination by high-performance size exclusion chromatography (HPSEC) using a TSK-gel G-3000 PW_XL_ column (7.8 × 300 mm) (Dou, Tian, Wang, et al., 2025). 1 mg of sample was mixed with 200 μL of 0.2 M NaCl solution, centrifuged (12,000 rpm, 10 min), and filtered through a 0.22 μm membrane. The filtrate was analyzed by high-performance gel permeation chromatography (HPGPC) with a TSK-gel G4000 PW_XL_ or TSK-gel G3000PW_XL_ column (7.8 × 300 mm, TOSOH, Japan) coupled to a Shimadzu LC-20Ai system. The elution was performed with 0.2 M NaCl at a flow rate of 0.6 mL/min, with the column temperature maintained at 40 °C. Standard curves were constructed using dextran standards of different molecular weights.

### 2.6 Monosaccharide composition analysis

Monosaccharide composition analysis was carried out as previously reported (Cui et al., 2020). Briefly, 1 mg of polysaccharide sample was mixed with 1 mL anhydrous methanol containing 2 M hydrochloric acid, reacted at 80 °C for 16 h, and then further reacted with 2 M trifluoroacetic acid at 120 °C for 1 h. Subsequently, the hydrolyzed sample was derivatized with 1-phenyl-3-methyl-5-pyrazolone (PMP), and the resulting derivatives were analyzed by using high-performance liquid chromatography (HPLC).

The monosaccharide composition was analyzed using a Shimadzu liquid chromatography system (LC-20 AT pump, autosampler, and SPD-20 A UV detector) equipped with a COSMOSIL 5C18-PAQ column (4.6 × 250 mm). The elution conditions were as follows: flow rate of 1 mL/min, column temperature maintained at 35 °C, and the mobile phase consisting of 0.1 M PBS (pH 7.0) and acetonitrile in a ratio of 81.5:18.5. The UV detection was performed at a wavelength of 245 nm, with an injection volume of 10 μL. The following monosaccharide standards were used, including mannose (Man), glucuronic acid (GlcA), rhamnose (Rha), galacturonic acid (GalA), glucose (Glc), galactose (Gal), xylose (Xyl), arabinose (Ara), and fucose (Fuc), which were derivatized by the same method as the samples.

### 2.7 UV and FT-IR analysis

Preliminary structural characterization of polysaccharides using spectroscopy (Dou, Tian, & Zhang, 2025). 4 mg of polysaccharide was weighed out and thoroughly mixed with 4 mL deionized water (dH_2_O). Using a full-wavelength UV–visible spectrophotometer (UV-2700, Shimadzu, Japan), a baseline was established with dH_2_O as the blank, followed by full-band scanning over the 190–900 nm range.

FT-IR was employed to characterize functional groups in polysaccharide samples (Mouille et al., 2010). Dried polysaccharide (1–2 mg) was weighed out and mixed with 150 mg of pre-dried spectroscopically pure potassium bromide (KBr). The mixture was thoroughly ground in a mortar for 10 min until a uniform fine powder was obtained. The powder was placed into a mold and pressed into a transparent thin sheet under 10 MPa pressure. The sheet was scanned on an infrared spectrometer (Spectrum Two, PE, America) over the 4000–400 cm^−1^ range, and functional groups were assessed based on the resulting infrared spectrum.

### 2.8 Methylation and GC–MS analysis

5 mg of sample was dissolved in 0.5 mL dimethyl sulfoxide (DMSO), followed by addition of 0.5 mL sodium hydroxide-DMSO suspension. Methylation was performed by adding 1 mL methyl iodide. The mixture was extracted with chloroform (CH_2_Cl_2_). The organic layer was collected, and solvent was removed by vacuum evaporation. Complete methylation was confirmed by the absence of the -OH stretching peak (3200–3400 cm^−1^) in FT-IR spectra. O-methylated polysaccharides were hydrolyzed sequentially with 1 mL 85% formic acid (100 °C, 4 h) and 1 mL 2 M formic acid (100 °C, 6 h). Partially methylated sugars were reduced with NaBH_4_, acetylated, and the resulting partially methylated alditol acetates were analyzed by GC–MS (7890B—5977B, Agilent, USA) using an HP-5 ms capillary column (30 m × 0.32 mm × 0.25 μm). The column temperature program was: 120 °C 1 min → 210 °C 2 min at 3 °C/min, then 260 °C 4 min at 10 °C/min. Inlet and detector temperatures were 300 °C with helium as the carrier gas. The scan range was 50–500 *m/z,* and the response factor was 1.00 (Qu et al., 2022).

### 2.9 Nuclear magnetic resonance spectroscopy (NMR)

^13^C NMR spectra were recorded on a Bruker Avance 600 MHz spectrometer (Karlsruhe, Germany) at 20 °C using a 5 mm broadband probe. 20 mg of sample was dissolved in 0.5 mL D_2_O or DMSO‑*d*_6_, centrifuged (12,000 rpm × 5 min) to remove precipitates, and the supernatant was analyzed. Data processing was performed using standard Bruker software (Zu et al., 2024).

## 3 Results

### 3.1 Sequential extraction of polysaccharides from *P. eryngii*

**Fig. 1:**
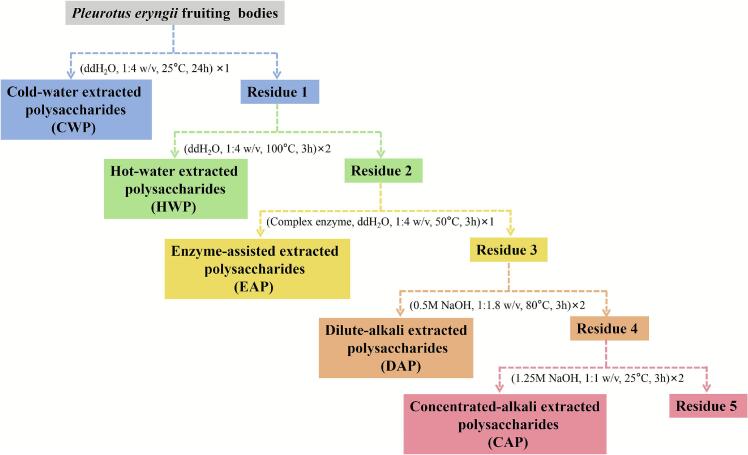
Sequential extraction flowchart of polysaccharides from *P. eryngii.*

To efficiently extract polysaccharides from *P. eryngii*, we employed a sequential extraction strategy comprised of cold-water extraction, hot-water extraction, enzyme-assisted extraction, dilute-alkali extraction, and concentrated-alkali extraction. The extraction process is illustrated in Fig. 1. Five polysaccharide fractions, namely CWP, HWP, EAP, DAP, and CAP, were obtained. The yield and composition of these polysaccharides are shown in Table 1. Our results show that the yields of polysaccharides CWP, HWP, and EAP are relatively similar, ranging from 0.10% to 0.19%, whereas DAP and CAP polysaccharide yields were higher at 0.65% and 0.34% respectively. With these enhanced extraction conditions, polysaccharide yields were significantly increased. The sugar content of CWP and HWP were relatively high (exceeding 80%), whereas sugar contents in fractions EAP, DAP, and CAP were 63%–75%. The uronic acid contents of the polysaccharides were relatively low (0.3%–1.8%), indicating that *P. eryngii* contains relatively low levels of acidic polysaccharides. The samples also contained minor proteins and ash, among which EAP had the highest content of protein (14.4%) and ash (9.9%). In summary, our sequential extraction process yielded total polysaccharide extractions of 1.42% (relative to the fresh fruiting body weight of *P. eryngii*), which was about fivefold higher compared to conventional hot water extraction (Zhang et al., 2020). Our approach enables the effective extraction of polysaccharides from *P. eryngii*.

**Table 1:** Yield and physicochemical properties of five polysaccharides.

Fractions	Yield (%)^a^	Yield (%)^b^	Sugar content (%)	Uronic Acid Content (%)	Protein content (%)	Ash content (%)
CWP	0.19	2.3	86.7	0.3	6.2	1.1
HWP	0.10	1.2	82.1	1.8	6.0	3.4
EAP	0.14	1.7	63.2	0.6	14.4	9.9
DAP	0.65	7.8	67.5	1.5	8.0	7.3
CAP	0.34	4.1	74.0	0.4	6.5	4.7

a Yield is based on the fresh weight of *P. eryngii* fruiting bodies.

b Yield is based on the dry weight of *P. eryngii* fruiting bodies*.* The ratio of fresh weight to dry weight of *P. eryngii* is determined as 12:1.

**Fig. 2:**
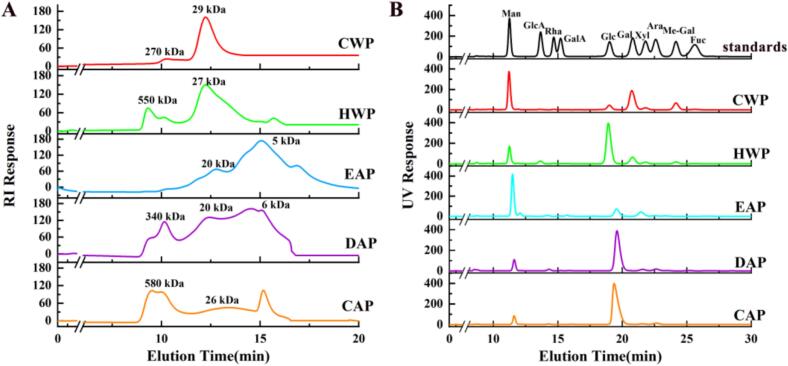
(A) Molecular weight distribution (TSK-gel G3000 PW_XL_) and (B) monosaccharide composition of five polysaccharides.

Molecular weight distributions of these polysaccharides were determined by using HPGPC. As shown in Fig. 2A, the molecular weight distribution of CWP was relatively simple, exhibiting a major peak, whereas other polysaccharides were heterogeneous. Monosaccharide composition analysis was performed to explore component compositions (Fig. 2B, Table 2). This showed that CWP is primarily composed of Man (39.9%), Gal (32.2%) and Me-Gal (10.7%), suggesting that CWP mainly contains galactans and mannans. HWP was primarily composed of Glc (57.9%), Man (17.5%), and Gal (14.7%), which was assumed to mainly contain glucan, along with a small amount of galactan. EAP is mainly composed of Man (62.7%). Both DAP and CAP are predominantly composed of Glc (>80%), indicating that these fractions mainly contain glucan. According to monosaccharide composition analysis, we could see specific types of polysaccharides can be selectively isolated during the sequential extraction protocol.

**Table 2:** Monosaccharide compositions of five polysaccharides.

Fractions	Monosaccharide composition (mol%)						
	Glc	Gal	Man	Me-Gal	Fuc	GlcA	Xyl
CWP	10.8	32.2	39.9	10.7	1.6	–	4.9
HWP	57.9	14.7	17.5	3.7	0.6	3.5	2.1
EAP	23.0	12.4	62.7	–	0.7	–	1.3
DAP	82.1	4.2	7.1	0.8	0.3	2.5	2.1
CAP	91.9	0.9	2.5	–	–	4.7	–

Monosaccharide abbreviations: Glc (Glucose), Gal (Galactose), Man (Mannose), Me-Gal (Methyl-galactose), Fuc (Fucose), GlcA (Glucuronic acid), Xyl (Xylose).

### 3.2 Purification of polysaccharides from *P. eryngii*

**Fig. 3:**
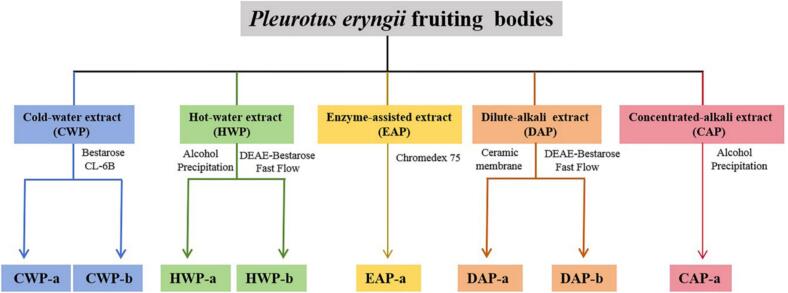
Flowchart for the purification of polysaccharides from *P. eryngii*.

Different methods were employed to isolate and purify the polysaccharides from *P. eryngii*, as shown in Fig. 3. CWP was separated by gel filtration chromatography, yielding two fractions: CWP-a (yield 15%) and CWP-b (yield 62%). HWP was separated by ethanol precipitation and ion-exchange chromatography, and two fractions HWP-a (yield 42%) and HWP-b (yield 25%) were obtained. EAP was separated by gel filtration chromatography to obtain a major fraction EAP-a, with a yield of 48%. DAP was fractionated by membrane filtration and ion-exchange chromatography, producing two fractions: DAP-a (yield 44%) and DAP-b (yield 28%). CAP was precipitated using 40% ethanol to get a major fraction CAP-a (yield 81%). Overall, a total of 8 polysaccharide fractions were obtained.

**Fig. 4:**
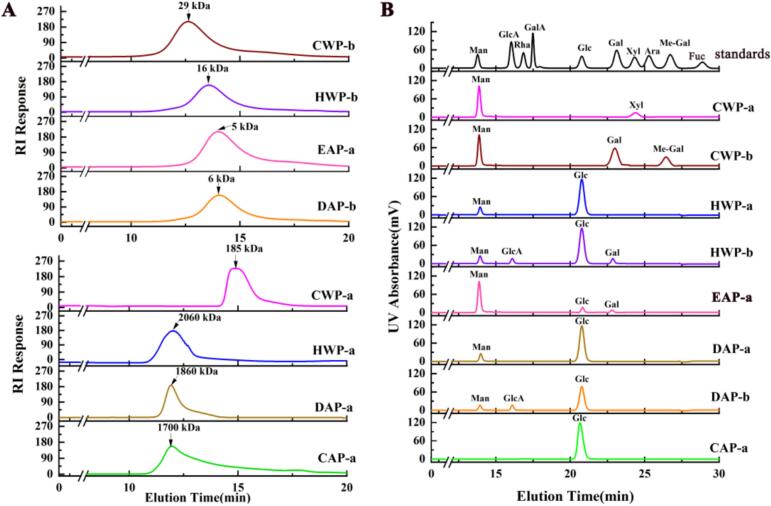
(A) Molecular weights determination (CWP-b, HWP-b, EAP-a, DAP-b were determined by TSK-gel G3000 PW_XL_, CWP-a, HWP-a, DAP-a, CAP-a were determined by TSK-gel G4000 PW_XL_). (B) Monosaccharide composition analysis.

The molecular weight and homogeneity of these purified fractions were determined by using HPGPC (Fig. 4A). CWP-b, HWP-b, EAP-a and DAP-b had relatively low molecular weights in the range of 29 kDa ∼ 5 kDa, whereas CWP-a, HWP-a, DAP-a, and CAP-a all had relatively high molecular weights in the range of 185 kDa ∼ 2060 kDa. The monosaccharide compositions of these fractions are shown in Fig. 4B and listed in Table 3. CWP-a mainly contains Man (58.6%) and Xyl (28.4%), indicating it is a xylomannan. CWP-b contains Gal (47.8%), Man (29.3%) and Me-Gal (17.4%) as major monosaccharides, suggesting it is a mannogalactan. EAP-a is a mannan, because it is mainly composed of Man (74.6%). HWP-a, HWP-b, DAP-a, DAP-b, and CAP-a each contain Glc (77% ∼ 97%) as the dominant monosaccharide, suggesting they are glucans, but may have different structures. Therefore, we speculated that polysaccharides from *P. eryngii* have diverse structures, including galactan, mannan and glucan.

**Table 3:** Monosaccharide compositions of polysaccharide fractions from *P. eryngii.*

Fractions	Monosaccharide composition (mol%)						
	Glc	Gal	Man	Me-Gal	Fuc	GlcA	Xyl
CWP-a	5.9	4.0	58.6	1.1	2.0	–	28.4
CWP-b	3.5	47.8	29.3	17.4	1.9	3.5	–
HWP-a	85.7	1.9	8.9	–	–	2.3	1.2
HWP-b	77.4	6.5	7.0	–	–	9.1	–
EAP-a	16.0	6.8	74.6	–	–	0.9	1.8
DAP-a	87.5	0.8	7.3	–	–	2.3	2.1
DAP-b	80.4	2.0	5.1	–	–	11.7	0.8
CAP-a	97.1	0.7	1.1	–	–	0.9	0.3

Monosaccharide abbreviations: Glc (Glucose), Gal (Galactose), Man (Mannose), Me-Gal (Methyl-galactose), Fuc (Fucose), GlcA (Glucuronic acid), Xyl (Xylose).

### 3.3 UV–VIS and FT-IR analysis of polysaccharide fractions from *P. eryngii*

**Fig. 5:**
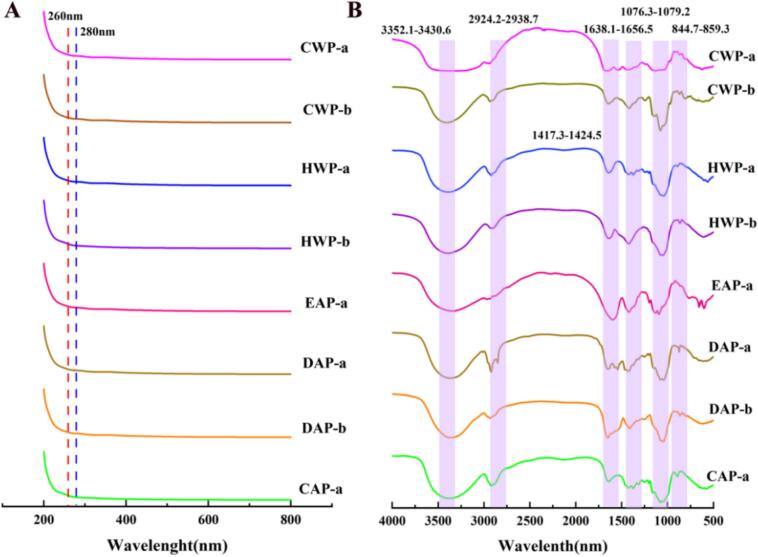
(A) UV–Vis and (B) FT-IR spectra of polysaccharide fractions from *P. eryngii.*

Polysaccharide fractions show no significant absorption peaks at 260 nm and 280 nm in UV–Vis spectral analysis (Fig. 5A), indicating that they contain negligible amounts of common impurities like nucleic acids and proteins.

FT-IR analysis (Fig. 5B) showed that the polysaccharide fractions exhibit strong absorption bands at 3403 cm^−1^and 3419 cm^−1^, attributable to O—H stretching vibrations arising from intermolecular hydrogen bonding interactions. Peaks near 2934 cm^−1^ and 2928 cm^−1^ are attributable to C—H stretching vibrations. Strong absorption bands at 1043 cm^−1^and 1068 cm^−1^ are characteristic of C-O-C bond vibrations. Characteristic peaks at 1642 cm^−1^ and 1645 cm^−1^ correspond to hydroxyl bending vibrations, and weak peaks at 1419 cm^−1^and 1412 cm^−1^ are assigned to C—H strain vibrations. In the fingerprint region, CWP-a and EAP-a showed an absorption peak at 803–807 cm^−1^, which are attributable to the α-configuration of sugar units. CWP-b exhibited characteristic absorption peaks at 805 cm^−1^ and 875 cm^−1^, which indicate both α- and β-configurations. HWP-a, HWP-b, DAP-a and DAP-b and CAP-a each display characteristic absorption peaks at around 865–875 cm^−1^, indicating β-configurations.

### 3.4 Methylation analysis of polysaccharide fractions from *P. eryngii*

**Fig. 6:**
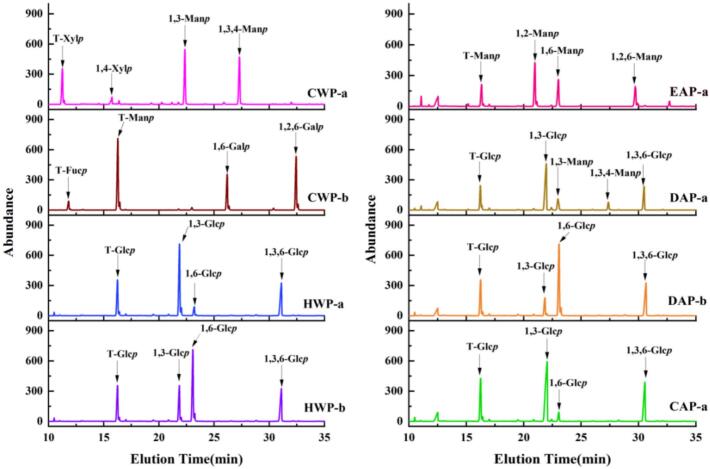
Methylation analysis of different polysaccharides.

Methylation analysis was performed to determine glycosidic linkage patterns (Fig. 6), and ion fragments are shown in Fig. S1. The glycosidic linkage types and proportions are listed in Table 4. CWP-a contains 1,3-Man*p* (35.3%) and 1,3,4-Man*p* (33.0%) as the major linkage types. Xylose residues are predominantly in the form of T-Xyl*p* (28.4%) with minor 1,4-Xyl*p* (3.2%). This indicates the backbone of this polysaccharide is formed by 1,3-Man*p*, and approximately 48.3% of the 1,3-Man*p* residues are branched at the O-4 position (degree of branching, DB = 48.3%), where T-Xyl*p* forms the primary side-chain structure with minor 1,4-Xyl*p* short side chains. Based on the linkage type of the backbone, CWP-a was classified as 1,3-D-mannan. EAP-a mainly contains 1,2-Man*p* (43.1%), together with 1,6-Man*p* (20.6%), 1,2,6-Man*p* (14.7%) and T-Man*p* (21.6%). This indicates that the polysaccharide backbone is primarily composed of 1,2-Man*p*, with approximately 25.4% of the backbone residues branching at the O-6 position (DB = 25.4%). The side chains might be short chains of 1,6-Man*p* and T-Man*p*. Therefore, EAP-a is classified as 1,2-D-mannan.

**Table 4:** Glycosidic linkage types and molar ratios in different polysaccharides.

Fractions	Methylated sugars	Linkages	Molar percentage (%)			Mass fragments (*m/z*)
			CWP-a			
1,3 -Mannan	2,4,6-Me_3_-Man*p*	1,3-	35.3			87,101,117,129,161,202,233
	2,6-Me_2_-Man*p*	1,3,4-	33.0			87,101,117,129,185,305
	2,3-Me_2_-Xyl*p*	1,4-	3.2			87,101,117,129, 189,233
	2,3,4-Me_3_-Xyl*p*	t-	28.5			87,101,117,129,145,161
**Fractions**	**Methylated sugars**	**Linkages**	**Molar percentage (%)**			**Mass fragments (*m/z*)**
			**EAP-a**			
1,2 -Mannan	2,3,4-Me_3_-Man*p*	1,6-	20.6			101,117,129,161,173,189,233
	3,4-Me_2_-Man*p*	1,2,6-	14.7			87,117,129,145,161,205
	3,4,6-Me_3_-Man*p*	1,2-	43.1			87,101,117,129,161,189,233
	2,3,4,6-Me_4_-Man*p*	t-	21.6			87,101,129,161,189
**Fractions**	**Methylated sugars**	**Linkages**	**Molar percentage (%)**			**Mass fragments (*m/z*)**
			**CWP-b**			
1,6 -Galactan	2,3,4-Me_3_-Gal*p*	1,6-	36.6			87,101,117,129,161,189,233
	3,4-Me_2_-Gal*p*	1,2,6-	31.5			71,87,99,129,189,233
	2,3,4,6-Me_4_-Fuc*p*	t-	1.2			72.89,101,117,131,161,233
	2,3,4,6-Me_4_-Man*p*	t-	36.7			71,89.101.117,129,161.205
**Fractions**	**Methylated sugars**	**Linkages**	**Molar percentage (%)**			**Mass fragments (*m/z*)**
			**HWP-a**	**DAP-a**	**CAP-a**	
1,3 -Glucan	2,3,4-Me_3_-Glc*p*	1,6-	4.8	–	4.6	101,117,129,161,173,189,233
	2,4-Me_2_-Glc*p*	1,3,6-	22.7	22.4	24.1	87,117,129,159,189,233
	2,4,6-Me_3_-Glc*p*	1,3-	53.0	53.6	50.0	71,87,101,117,129,161,233
	2,3,4,6-Me_4_-Glc*p*	t-	19.5	21.0	21.3	87,101,117,129,145,161,205
**Fractions**	**Methylated sugars**	**Linkages**	**Molar percentage (%)**			**Mass fragments (*m/z*)**
			**HWP-b**	**DAP-b**		
1,6 -Glucan	2,3,4-Me_3_-Glc*p*	1,6-	57.0	36.8		101,117,129,161,173,189,233
	2,4-Me_2_-Glc*p*	1,3,6-	14.8	23.5		87,101,117,129,159,189,233
	2,4,6-Me_3_-Glc*p*	1,3-	13.6	12.8		87,101,117,129,161,189,233
	2,3,4,6-Me_4_-Glc*p*	t-	14.6	26.9		87,101,117,129,145,161,205

CWP-b contains 1,6-Gal*p* (36.6%) as the primary linkage, followed by 1,2,6-Gal*p* (31.5%), which suggests that the backbone of this polysaccharide is composed of 1,6-Gal*p*, with approximately 46.3% of backbone residues being branched at the O-2 position (DB = 46.3%). The side chain in CWP-b is T-Man*p* (30.7%). Methylation analysis confirmed that CWP-b is a 1,6-D-galactan.

HWP-a, DAP-a, and CAP-a each contains 1,3-Glc*p* as the major glycosidic linkage, with molar ratios of 53.0%, 50.6% and 50.0%, respectively. In addition, these polysaccharides contain nearly equivalent ratios of 1,3,6-Glc*p* (19.4% ∼ 24.4%) and T-Glc*p* (18.0% ∼ 21.3%). Our results suggest that these polysaccharides are 1,3-D-glucans, that have a backbone composed of 1,3-Glc*p* units branched at the O-6 position with T-Glc*p*. The DBs of these are 30.0%, 27.9% and 32.9%. HWP-b and DAP-b are each composed of 1,6-Glc*p* as the major glycosidic linkage, with molar ratios of 57.0% and 36.8%, respectively. Meanwhile, each contains 1,3,6-Glc*p* (14.8%, 23.5%), 1,3-Glc*p* (13.6%, 12.8%) and T-Glc*p* (14.6%, 26.9%). Therefore, they are both 1,6-D-glucans, with backbones that consist of 1,6-Glc*p* units with branching at the O-3 with 1,3-Glc*p* and T-Glc*p* side chains. The DBs of these are 20.6% and 39.0%, respectively, suggesting that DAP-b has more side chains than HWP-b.

### 3.5 NMR analysis of polysaccharide fractions from *P. eryngii*

Based on methylation analysis, we identified five types of polysaccharides, including 1,3-D-mannan, 1,2-D-mannan, 1,6-D-galactan, 1,3-D-glucan and 1,6-D-glucan. To further characterize structural features, representative polysaccharide were analyzed by NMR spectroscopy.

**Fig. 7:**
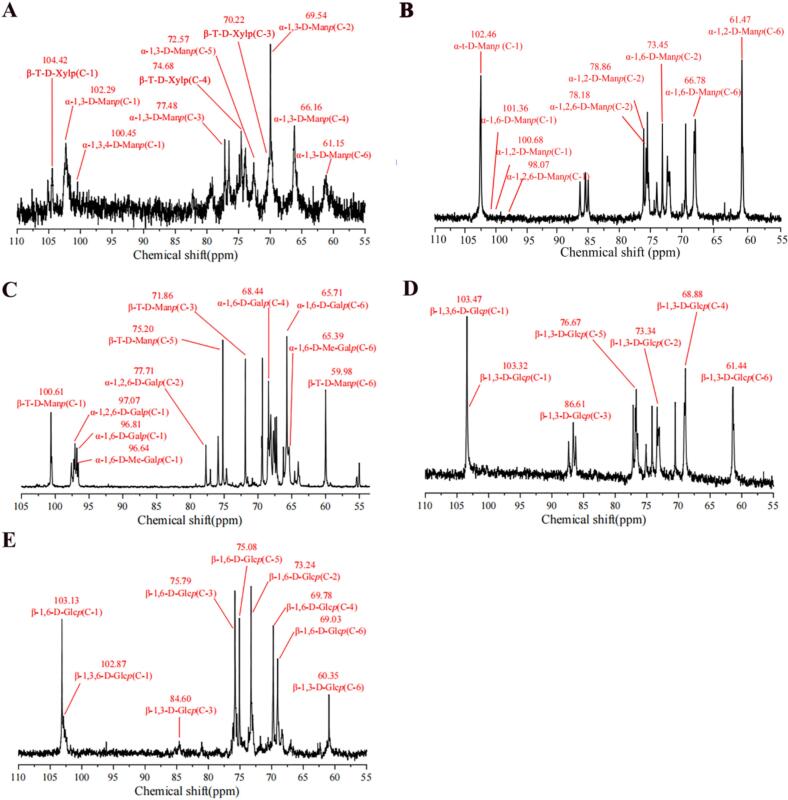
^13^C NMR analysis of (A) α-1,3-Mannan (B) α-1,2-Mannan (C) α-1,6-Galactan (D) β-1,3-Glucan and (E) β-1,6-Glucan.

NMR analysis of CWP-a (Fig. 7A) showed signals at 104.42 ppm, 102.29 ppm, 100.45 ppm, which correspond to the C-1 of β-T-D-Xyl*p*, α-1,3-D-Man*p* and α-1,3,4-D-Man*p*, respectively. Chemical shifts at 77.48 ppm, 72.57 ppm, 69.54 ppm, 66.16 ppm and 61.15 ppm could be assigned to C-3, C-5, C-2, C-4 and C-6 of α-1,3-D-Man*p*. Signals at 74.68 ppm and 70.22 ppm are attributed to C-4 and C-3 of β-T-D-Xyl*p*. respectively. In combination with methylation analysis, we determined that CWP-a is an α-1,3-D-mannan, with its main chain composed of α-1,3-Man*p* units branched at the O-4 primarily with β-T-Xyl*p* side chains.

NMR analysis of EAP-a (Fig. 7B) shows signals at 102.46 ppm, 101.36 ppm, 100.68 ppm and 98.07 ppm, which correspond to the C-1 resonances of α-T-D-Man*p*, α-1,6-D-Man*p*, α-1,2-D-Man*p* and α-1,2,6-D-Man*p*, respectively. Chemical shifts at 78.18 ppm can be assigned to C-2 of α-1,2-D-Man*p*, and the signal at 73.45 ppm is assigned to C-2 of α-1,6-D-Man*p*. Chemical shifts at 66.78 ppm and 67.04 ppm are attributed to C-6 of α-1,2,6-D-Man*p* and α-1,6-D-Man*p*, respectively (Yang et al., 2025). Therefore, EAP-a is an α-1,2-D-mannan, with its backbone consisting of α-1,2-Man*p* units branched at the O-6 position of backbone residues. The side chains are composed of α-T-Man*p* and α-1,6-Man*p* units. As α-1,2-D-mannan is a novel type of polysaccharide found in *P. eryngii*, the structure of it was confirmed by HSQC and HMBC spectrum (Fig. S2 and Table S1).

In NMR spectra of CWP-b (Fig. 7C), signals at 100.61 ppm, 97.07 ppm, 96.81 ppm and 96.64 ppm are attributed to C-1 of β-T-Man*p*, α-1,2,6-D-Gal*p*, α-1,6-D-Gal*p* and α-1,6-D-Me-Gal*p*, respectively. Chemical shifts at 65.71–65.39 ppm are assigned to C-6 of α-1,6-D-Gal*p*, α-1,2,6-D-Gal*p* and α-1,6-D-Me-Gal*p*. The peak at 77.71 ppm can be assigned to C-2 of α-1,2,6-D-Gal*p*. The signal at 54.96 ppm confirmed the existence of -OCH_3_ groups, and the signal at 77.70 ppm is assigned to O-3 of α-1,6-D-Me-Gal*p*. Chemical shifts of 75.20 ppm, 71.86 ppm and 59.98 ppm are attributed to C-5, C-3 and C-6 of β-T-Man*p* (Wang et al., 2014). These results indicate that CWP-b is an α-1,6-D-galactan, with its backbone consisting of α-1,6-D-Gal*p* units along with some Gal units having methyl groups at the O-3 position. Branching occurs at the O-2 position of backbone residues, with side chains of β-T-Man*p*.

HWP-a was selected as a representative 1,3-glucan for NMR analysis. In the ^13^C NMR spectrum (Fig. 7D), signals at 103.47 ppm, 103.32 ppm and 103.91 ppm are attributed to C-1 resonances of β-1,3,6-D-Glc*p*, β-1,3-D-Glc*p* and β-T-D-Glc*p*, respectively. Signals at 86.61 ppm and 87.13 ppm are assigned to C-3 of β-1,3-D-Glc*p* and β-1,3,6-D-Glc*p*. The signal at 68.13 ppm was assigned to C-6 of β-1,3,6-D-Glc*p* (Luo et al., 2025). Chemical shifts for C-1 to C-6 of these sugar residues are listed in Table 5. Combined with methylation analysis, we concluded that HWP-a is a β-1,3-D-glucan, with its main chain composed of β-1,3-Glc*p*, and β-T-Glc*p* side chains linked to the O-6 of main chain Glc residues.

**Table 5:** NMR chemical shift assignments of different types of polysaccharides.

Linkage type (CWP-a)	Chemical shift(ppm)					
	C-1	C-2	C-3	C-4	C-5	C-6
→3)-α-D-Man*p*-(1→	102.29	69.54	77.48	66.16	72.57	61.15
→3,4)-α-D-Man*p*-(1→	100.45	68.42	78.46	69.21	73.12	60.41
β-D-Xyl*p*-(1→	104.42	72.56	70.22	74.68	66.14	–
Linkage type (EAP-a)	Chemical shift(ppm)					
	C-1	C-2	C-3	C-4	C-5	C-6
→2)-α-D-Man*p*-(1→	100.68	78.86	70.12	67.05	73.38	61.47
→2,6)-α-D-Man*p*-(1→	98.07	78.18	71.28	66.61	73.40	66.78
→6)-α-D-Man*p*-(1→	101.36	73.45	71.90	68.50	72.53	67.04
α-D-Man*p*-(1→	102.46	71.26	67.10	73.90	71.24	62.30
Linkage type (CWP-b)	Chemical shift(ppm)					
	C-1	C-2	C-3	C-4	C-5	C-6
→6)-α-D-Gal*p*-(1→	96.81	67.86	67.32	68.44	66.94	65.71
→6)-α-D-Me-Gal*p*-(1→	96.64	66.20	77.69	64.01	67.74	65.39
→2,6)-α-D-Gal*p*-(1→	97.07	77.71	67.22	67.40	67.71	65.56
β-D-Man*p*-(1→	100.61	70.34	71.86	69.31	75.20	59.98
Linkage type (HWP-a)	Chemical shift(ppm)					
	C-1	C-2	C-3	C-4	C-5	C-6
→3)-β-D-Glc*p*-(1→	103.32	73.34	86.61	68.88	76.67	61.44
→3,6)-β-D-Glc*p*-(1→	103.47	72.31	87.13	68.44	74.88	68.13
β-D-Glc*p*-(1→	103.91	72.07	74.50	67.73	73.70	61.49
Linkage type (HWP-b)	Chemical shift(ppm)					
	C-1	C-2	C-3	C-4	C-5	C-6
→6)-β-D-Glc*p*-(1→	103.13	73.24	75.79	69.78	75.08	69.03
→3,6)-β-D-Glc*p*-(1→	102.87	73.16	84.17	69.79	75.08	68.86
→3)-β-D-Glc*p*-(1→	102.67	72.73	84.60	68.40	74.86	60.35
β-D-Glc*p*-(1→	102.56	72.56	73.26	68.54	73.38	60.48

HWP-b was selected as a representative 1,6-glucan for NMR analysis. In the ^13^C NMR spectrum (Fig. 7E), six major signals at 103.13 ppm, 73.42 ppm, 75.79 ppm, 69.78 ppm, 75.08 ppm and 69.03 ppm could be assigned to C-1, C-2, C-3, C-4, C-5 and C-6 of β-1,6-D-Glc*p*. Chemical shifts at 102.87 ppm, 84.17 ppm and 68.86 ppm correspond to C-1, C-3 and C-6 of β-1,3,6-D-Glc*p*. Chemical shifts at 102.67 ppm and 84.60 ppm are assigned to C-1 and C-3 of β-1,3-D-Glc*p* (Tondolo et al., 2017). The corresponding chemical shifts for β-T-D-Glc*p* were also assigned. Accordingly, HWP-b is a β-1,6-D-glucan with its backbone consisting of β-1,6-Glc*p* units branched at the O-3 position with β-1,3-Glc*p* and β-T-Glc*p* units.

### 3.6 Structural models and contents of polysaccharides from *P. eryngii*

**Fig. 8:**
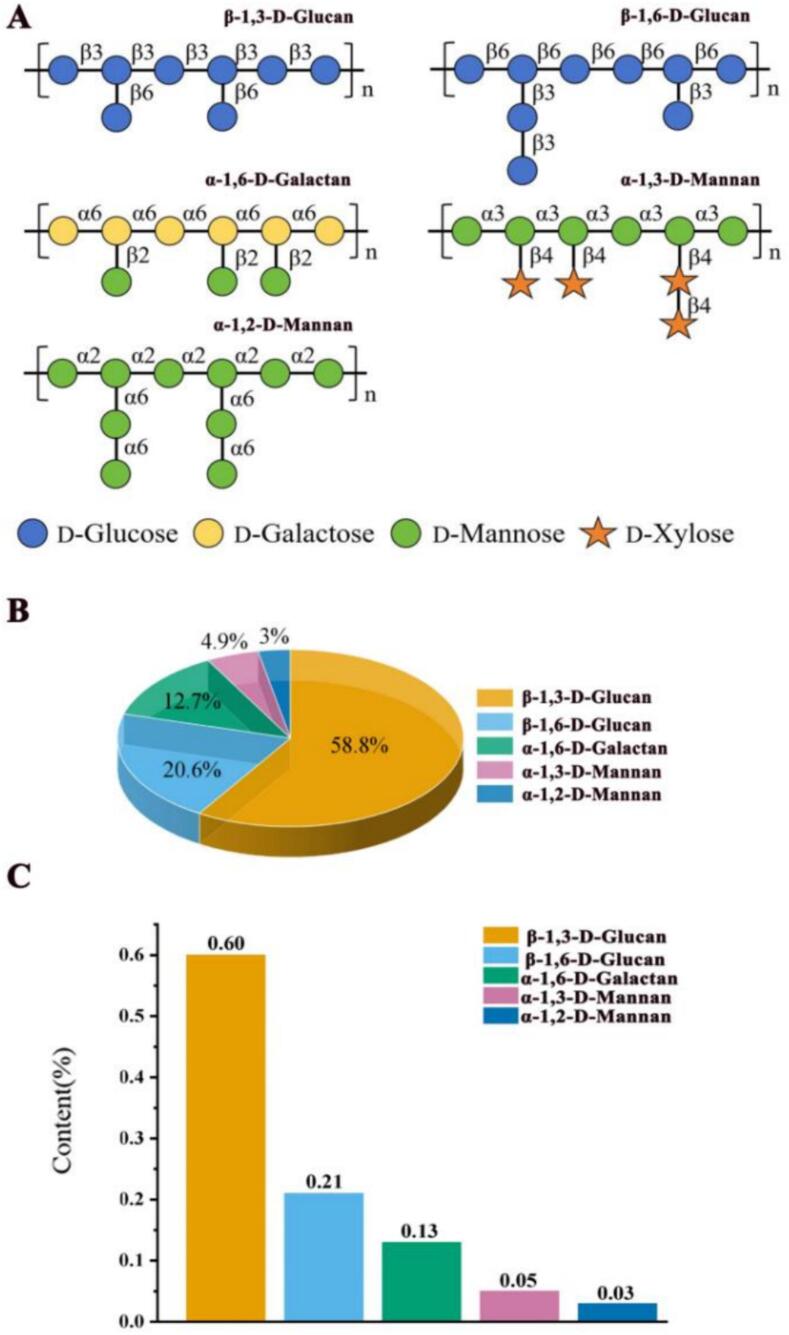
(A) Structural models of polysaccharides from *P. eryngii*. (B) Proportion of five types of polysaccharides in the total amount of polysaccharides. (C) Proportion of five types of polysaccharides compared with the fresh *P. eryngii* fruiting bodies.

Based on monosaccharide composition analysis, FT-IR, methylation analysis, and NMR results, the structures of eight polysaccharide fractions from *P. eryngii* could be classified into five types (Fig. 8A): β-1,3-D-glucan, β-1,6-D-glucan, α-1,6-D-galactan, α-1,3-D-mannan, and α-1,2-D-mannan. Among these fractions, HWP-a, DAP-a, and CAP-a are all β-1,3-D-glucans with backbones composed of β-1,3-D-Glc*p* units branched at the O-6 position, primarily with β-T-Glc*p*. The branching degrees of HWP-a, DAP-a, and CAP-a are 30.0%, 27.9%, and 32.9%, respectively. HWP-b and DAP-b are both β-1,6-D-glucans with backbones composed of β-1,6-D-Glc*p* units branched at the O-3 position with short chains of β-1,3-D-Glc*p* and β-T-Glc*p* units and branching degrees of 20.6% and 39.0%, respectively. CWP-b is an α-1,6-D-galactan, with a backbone composed of α-1,6-D-Gal*p* units. Branching occurs at the O-2 position with side chains of β-T-Man*p* units and a branching degree of 46.3%. CWP-a is an α-1,3-D-mannan with a backbone consisting of α-1,3-Man*p* units and side chains linked to the O-4 position by β-T-Xyl*p* and minor β-1,4-D-Xyl*p* and a branching degree of 48.3%. EAP-a is an α-1,2-D-mannan with a backbone of α-1,2-Man*p* units branched at the O-6 position with α-1,6-Man*p* and α-T-Man*p* units and a branching degree of 25.4%. To our knowledge, α-1,2-D-mannan is the first mannan of this type isolated from *P. eryngii.*

The proportions of five types of polysaccharides in the total polysaccharide content from *P. eryngii* is shown in Fig. 8B. The proportion of β-1,3-D-glucan, β-1,6-D-glucan, α-1,6-D-galactan, α-1,3-D-mannan and α-1,2-D-mannan is 58.8%, 20.6%, 12.7%, 4.9% and 3.0%, respectively. Compared with the fresh *P. eryngii* fruiting bodies (Fig. 8C), the ratio is 0.60%, 0.21%, 0.13%, 0.05% and 0.03%, respectively. Therefore, β-1,3-D-glucan is the dominant polysaccharide in *P. eryngii*, whereas α-1,2-D-mannan has the lowest content.

## 4 Discussion

At present, different methods have been used for extraction of polysaccharides from *P. eryngii.* For example, hot water extraction was performed to extract *P. eryngii* polysaccharides, with a yield of 7.46 ± 0.1% (g/100 g dried weight of *P. eryngii*) (Chen et al., 2013). Alkali extraction was used to obtain *P. eryngii* polysaccharides with the yield of 6.3% (g/100 g dried weight of *P. eryngii*) (Hao et al., 2017). Ultrasonic extraction yielded *P. eryngii* polysaccharides with a yield of 7.5 ± 0.3% (g/100 g dried weight of *P. eryngii*) (Liu et al., 2010). However, single extraction methods could not sufficiently extract polysaccharides and result in relatively low recovery from *P. eryngii*. To address this, this study developed a sequential extraction protocol with gradient enhancement of extraction conditions. We first extracted free polysaccharides or outer layer polysaccharides of the cell wall from *P. eryngii* under mild water/enzyme conditions, followed by enhanced conditions under dilute/concentrated alkali to extract insoluble polysaccharides in the inner layer of cell wall. With the sequential extraction strategy, the total extraction yield reaching 1.42% of the fresh *P. eryngii* fruiting bodies, equivalent to 17.1% of the dry weight. Therefore, the sequential extraction strategy developed here is practical, which significantly improving the extraction yield compared to conventional methods.

Previous studies have shown that *P. eryngii* contains branched β-1,3/1,6-glucan (Synytsya et al., 2009). Partially 3-O-methylated α-1,6-galactan was identified, with a molar ratio of galactose to 3-O-methyl-galactose of 3:1 (Carbonero et al., 2008). Additionally, a mannan was also identified with a backbone composed of α-1,3-man*p* units, primarily bearing β-t-xyl*p* side chains at the O-4 position (Zhao et al., 2025). However, there is still a lack of comprehensive analysis of polysaccharides from *P. eryngii.* It is not clear whether other types of polysaccharides exist in *P. eryngii.* In addition, the relative abundance and ratio of different types of polysaccharides in *P. eryngii* are also unclear. In this study, through sequential extraction and comprehensive structural elucidation, five distinct polysaccharide types including β-1,3-glucan, β-1,6-glucan, α-1,6-galactan, α-1,3-mannan and α-1,2-mannan were characterized in *P. eryngii,* among which α-1,2-mannan was first detected. The proportion of five types of polysaccharides is 20:7:4.3:1.6:1. We also found that different extraction methods could selectively extract specific types of polysaccharides to a certain extent. For example, cold water could extract α-1,3-mannan and α-1,6-galactan, enzyme-assisted method selectively extract α-1,2-mannan, while alkali extraction primarily obtain β-1,3-glucan and β-1,6-glucan.

Polysaccharides from *P. eryngii* have multiple biological activities, such as antitumor effects that inhibit tumor cell proliferation and metastasis (Ellefsen et al., 2023), lipid-lowering actions that regulate lipid metabolism pathways and hypoglycemic effects that competitively inhibit α-glucosidase activity (Zheng et al., 2019). Glucans from *P. eryngii* have been reported to have antioxidant functions that scavenge reactive oxygen species (Li et al., 2016). Galactans from *P. eryngii* could promote the production of NO, TNF-α, IL-6, and IL-1β, thereby activating macrophages and enhancing their phagocytic ability, exerting immunomodulatory activity (Yan et al., 2019). The current study primarily focuses on the establishment of a sequential extraction strategy and comprehensive analysis of polysaccharides from *P. eryngii*, thus does not carry out biological activity evaluation of these polysaccharides. In future work, we plan to systematically investigate their bioactivities, and deeply discuss the structure-activity relationships. These studies will provide a theoretical basis for developing and utilizing of polysaccharides from *P. eryngii*.

## 5 Conclusions

In this study, we developed a sequential extraction strategy to purify polysaccharides from fruiting bodies of *P. eryngii*. Using cold water, hot water, enzymatic-assisted, dilute alkali and concentrated alkali extraction, we report the total yield of polysaccharides as 1.42% of the fresh *P. eryngii* fruiting bodies. Following purification, we obtained homogeneous polysaccharide fractions and structurally analyzed them. *P. eryngii* polysaccharides consist of five types: β-1,3-D-glucan (58.8%), β-1,6-D-glucan (20.6%), α-1,6-D-galactan (12.7%), α-1,3-D-mannan (4.9%), and α-1,2-D-mannan (3.0%), with molar ratios of 20:7:4.3:1.6:1. Our study has systematically investigated the structural types and contents of polysaccharides in *P. eryngii*, and we demonstrated that different types of polysaccharides could be selectively extracted by using these extraction conditions. As anticipated, the sequential extraction approach not only enhances polysaccharide extraction efficiency, but it also enables preliminary separation of polysaccharides during processing. Consequently, our results provide useful information for further structure-activity relationships study of polysaccharides from *P. eryngii*.

## CRediT authorship contribution statement

**Ziyan Sun:** Writing – original draft. **Zhicong Song:** Formal analysis. **Andong Zhou:** Formal analysis. **Yongjian Lin:** Formal analysis. **Jiahui Yao:** Formal analysis. **Kevin H. Mayo:** Writing – review & editing. **Yifa Zhou:** Writing – review & editing. **Lin Sun:** Supervision.

## Declaration of competing interest

The authors declare that they have no known competing financial interests or personal relationships that could have appeared to influence the work reported in this paper.

## Data Availability

Data will be made available on request.

## Appendix A Supplementary data

Supplementary material Image 1
