# Supplementary material for: Polysaccharides from Pleurotus eryngii: sequential extraction and structural characterization
Source: Food Chem X. 2026 Jan 20;34:103543. doi: 10.1016/j.fochx.2026.103543 (PMC12860377; doi:10.1016/j.fochx.2026.103543)
Supplement: Supplementary file 1 — Supplementary material [file mmc1.docx]

**Supporting Information**

**Polysaccharides from Pleurotus eryngii: Sequential extraction and structural characterization**

Ziyan Sun^1^, Zhicong Song^1^, Andong Zhou^1^, Yongjian Lin^1^, Jiahui Yao^1^, Kevin H. Mayo^2^, Yifa Zhou^1^, Lin Sun^1,^*

^1^Engineering Research Center of Glycoconjugates of Ministry of Education, Jilin Provincial Key Laboratory of Chemistry and Biology of Changbai Mountain Natural Drugs, School of Life Sciences, Northeast Normal University, Changchun 130024, China

^2^Department of Biochemistry, Molecular Biology & Biophysics, University of Minnesota, 6-155 Jackson Hall, Minneapolis, MN 55455, USA

* Corresponding authors.

1. mail addresses: [sunl925@nenu.edu.cn](mailto:zhouyf383@nenu.edu.cn)

Content

[Supplementary Figures 1](#_Toc1102201879)

[Figure S1. Methylated Ion Fragment Peaks of eight polysaccharides 2](#_Toc1945117732)

[Figure S2. HSQC and HMBC analysis of α-1,2-D-mannan 3](#_Toc1945117732)

[Table S1. HSQC spectral assignments of α-1,2-D-mannan 3](#_Toc1945117732)





Figure S1. Methylated Ion Fragment Peaks of Each Polysaccharide Fractions

1. CWP-a. (B) CWP-b. (C) HWP-a. (D) HWP-b. (E) EWP-a. (F) DAP-a. (G) DAP-b. (H) CAP-a.


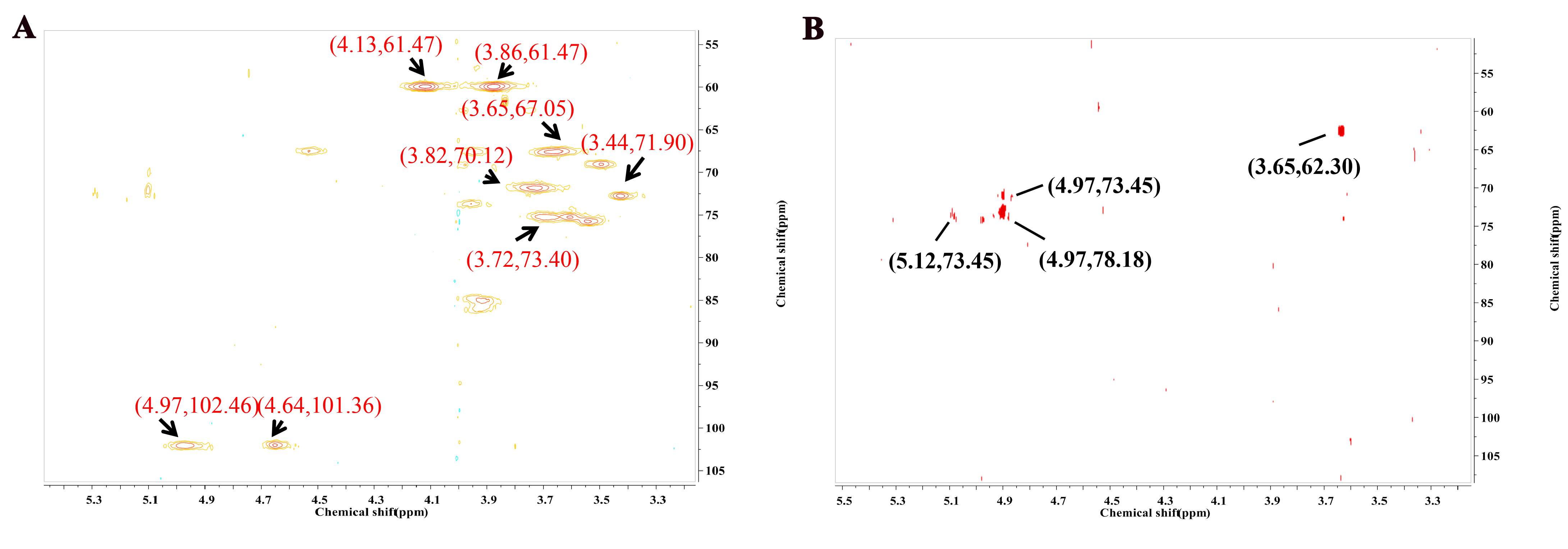
Figgure S2. NMR analysis of α-1,2-D-mannan (A) HSQC; (B) HMBC

Table S1. HSQC spectral assignments of α-1,2-D-mannan

| Linkage type | Chemical shift（ppm） | | | | | |
| --- | --- | --- | --- | --- | --- | --- |
|  | H-1  C-1 | H-2  C-2 | H-3  C-3 | H-4  C-4 | H-5  C-5 | H-6a;6b  C-6 |
| (A)→2)-α-D-Man*p*-(1→ | 5.12 | 4.03 | 3.82 | 3.65 | 3.68 | 4.13；3.81 |
|  | 100.68 | 78.86 | 70.12 | 67.05 | 73.38 | 61.47 |
| 1. →2,6)-α-D-Man*p*-(1→ | 5.02 | 3.95 | 3.75 | 3.64 | 3.72 | 3.93；3.58 |
|  | 98.07 | 78.18 | 71.28 | 66.61 | 73.40 | 66.78 |
| 1. →6)-α-D-Man*p*-(1→ | 4.64 | 3.95 | 3.44 | 3.63 | 3.79 | 3.52；3.78 |
|  | 101.36 | 73.45 | 71.90 | 68.50 | 72.53 | 67.04 |
| 1. α-D-Man*p*-(1→ | 4.97 | 3.98 | 3.75 | 3.56 | 3.68 | 3.81；3.67 |
|  | 102.46 | 71.26 | 67.10 | 73.90 | 71.24 | 62.30 |
